# High-Temperature-Induced Shape Memory Copolyimide

**DOI:** 10.3390/polym13193222

**Published:** 2021-09-23

**Authors:** Yucheng Zi, Dongxu Pei, Jianhua Wang, Shengli Qi, Guofeng Tian, Dezhen Wu

**Affiliations:** State Key Laboratory of Chemical Resource Engineering, Beijing University of Chemical Technology, Beijing 100029, China; ziyc1996@163.com (Y.Z.); peipeidongxu@163.com (D.P.); wjh444252009@163.com (J.W.); qisl@mail.buct.edu.cn (S.Q.); wdz@mail.buct.edu.cn (D.W.)

**Keywords:** polyimide film, copolymerization, high-temperature-induced, shape memory effect

## Abstract

A series of polyimide (PI) films with a high-temperature-induced shape memory effect and tunable properties were prepared via the facile random copolymerization of 4,4′-oxydianiline (ODA) with 4,4′-(hexafluoroisopropyl)diphthalic anhydride (6FDA) and 4,4′-oxydiphthalic anhydride (ODPA). The trigger temperature can be controlled from 294 to 326 °C by adjusting the ratio of monomers. The effects of monomer rigidity on the chain mobility, physical properties, and shape memory performance of as-prepared copolyimide were systematically investigated. The introduction of ODPA could enhance the mobility of PI macromolecular chains, which made chain entanglement more likely to occur and increased the physical crosslinking density, thereby improving the PI’s shape recovery up to 97%. Meanwhile, the existence of 6FDA enabled PI films to set quickly at low temperatures with a shape fixation of 98%. The shape memory cycling characteristics of the polyimide films are also studied, and the relationship between the PI chemical structure and the film properties are further discussed.

## 1. Introduction

Shape memory polymers (SMPs) with heat resistance and high-temperature responses have found broad applications in high-temperature sensors and actuators, aerospace self-deployable structures, and smart jet propulsion systems [1]. Aromatic polyimides (PIs), thanks to their unique aromatic heterocyclic rigid structure, possess excellent thermal, mechanical, and insulative properties, as well as outstanding resistance to space radiation. However, in various forms such as films [2], fibers [3], and composite materials [4], they have played essential roles in the aerospace industry [5]. Outstanding comprehensive performance [6] and structural designability [7,8] enable deformed PI materials to recover their initial shape under high-temperature conditions [9], which endows SMPs with more potential applications as expandable structures [10] such as solar arrays and expandable panels [11,12].

The thermally induced shape memory effect requires PI materials to possess a crosslinked structure [13]. Thermoplastic materials only have secondary bonds (hydrogenic, van der Waals, polar) or physical crosslinks among the molecular chains [14], but thermosets, in addition to having secondary bonds, have primary (covalent) bonds between their molecular chains [15]. Due to the existence of crosslinking points, changing a material’s shape by drafting or folding at temperatures higher than its glass transition temperature merely deviates chain segments from the equilibrium, instead of leading an entire chain to slip. Then, lowering the temperature to freeze the chain segments fixes the material’s shape. When heating the material above the glass transition temperature, entropic elasticity allows the chain segments to return to the equilibrium position under the action of the crosslinking network so as to trigger the shape recoveries [16,17]. For instance, Kong and Xiao [18] added ether bonds and flexible segments to the molecular chain by simultaneously employing 4,4′-(1,1′-biphenyl-4,4′-diyldioxy)dianiline (BAPB) and bis phenol A dianhydride (BPADA) to provide the macromolecules with a large number of entanglements and strong π–π interactions between the chains that function as a stationary phase. The PI film exhibited excellent shape memory recycling performance. Xiao et al. [19] added TAP to the 6FDA-ODA system as a chemical crosslinker. The chemically crosslinked product showed a higher T_g_ and better shape memory performance.

Shape fixity (R_f_) and shape recovery (R_r_) are two critical parameters of shape memory polymers, which represent an SMP’s ability to maintain mechanical deformation and return to its original shape, respectively. The rapid decrease of an SMP’s storage modulus in a small temperature range ensures an outstanding R_f_ [20,21], and the molecular chains’ entanglement and physical crosslinking lead to a desirable R_r_. However, the difficulty in shape maintenance after high-temperature deformation makes it challenging to enhance R_f_ and R_r_ at the same time [22,23]. Given that the 6FDA-ODA system with a sharp tan delta peak displays a sharp storage modulus drop near the glass transition temperature, while the ODPA-ODA system contains a large number of ether bonds (which is conducive to entanglement and therefore a better shape recovery), we herein prepared a series of copolyimide materials by integrating the above two systems. Taking advantage of the characteristics of both, we successfully developed an SMP film that could balance shape fixity rate and shape recovery rate.

## 2. Materials and Work Method

### 2.1. Materials

We purchased 4,4′-Oxydiphthalic dianhydride (ODPA) from Shanghai Saen Chemical Technology Co., Ltd. (Shanghai, China). 4,4′-(hexafluoroisopropylidene) diphthalic anhydride (6FDA) was procured from Shanghai Maclean Biochemical Technology Co., Ltd. (Shanghai, China). Beijing Forsman Technology Co., Ltd. (Beijing, China) provided 4,4′-oxydianiline (ODA). N,N-dimethylacetamide (DMAc) was purchased from Tianjin Fuchen Chemical Reagent Factory (Tianjin, China).

### 2.2. Preparation of Copolymer PI Film

**Scheme 1:**
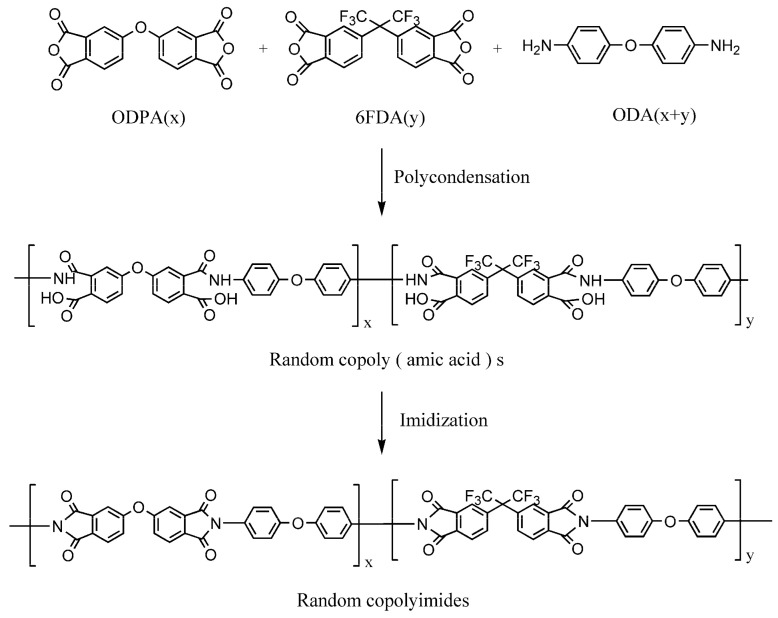
Reaction mechanism of 6FDA/ODPA-ODA PI.

The random copolyimides were synthesized from ODA, 6FDA, and ODPA via a two-step method. The molar ratio of dianhydride to diamine was 1.01:1, and the theoretical relative molecular masses ranged from 40,000 to 60,000. Briefly, ODA was added into a three-neck flask, followed by DMAc, which was continuously stirred under nitrogen purge until the ODA was completely dissolved. Then, 6FDA and ODPA were added into the solution in batches. The mixture was stirred for 4 h at a low temperature to yield a viscous poly (amic acid) (PAA) solution. The homogeneous PAA solution obtained was spread on a cleaned glass plate and then successively imidized at 60 °C for 2 h, 135 °C for 2 h, and 300 °C for 2 h. According to the different copolymerization ratios of the two dianhydrides, the obtained PI films were named 6FDA-ODA, 6FDA/ODPA-ODA-73, 6FDA/ODPA-ODA-55, 6FDA/ODPA-ODA-37, and ODPA-ODA. In the case of 6FDA/ODPA-ODA-73, the molar ratio of 6FDA to ODPA is 7:3. The synthetic route to the 6FDA/ODPA-ODA system is shown in Scheme 1.

### 2.3. Characterizations

FT-IR measurements were performed on a Nexus 670, made by Nicolet Instruments (Madison, WI, USA), with the scanning wavenumbers ranging from 4000 to 400 cm^−1^ to characterize whether the PI was successfully prepared.

Dynamic mechanical thermal analyses (DMA) of films were measured with a TA-Q800, made by TA Instruments, to evaluate the storage modulus and the tan delta of PI films. The glass transition temperature T_g_ can be also measures by the inflection point of the storage modulus or the maximum peak of the tan delta. These values are important to fix the process temperature in the shape memory step. DMA tests were performed in the temperature range between 50 and 350 °C, under nitrogen flux and with a heating rate of 5 °C/min. The sample of PI film was cut into long splines with a length of 30 mm and a width of 5.27 mm.

Thermogravimetric analysis (TGA) was performed under nitrogen atmosphere on a Setline Sta instrument made by Setaram company. The TGA test was performed with a heating rate of 10 °C/min from 30 °C to 800 °C.

The mechanical properties of PI films were tested at room temperature on a SANS CMT 4104 tensile apparatus made by MTS company with dumbbell-shaped specimens and with a crosshead speed of 10 mm/min in accordance with GB/T1040.3-2006 [24].

### 2.4. Shape Memory Performance Test

The shape memory properties of all samples were quantitatively assessed by DMA using a tensile film fixture and a controlled force mode to measure the deformation and recovery of the film shape.

At DMA’s control force mode, a spline was heated to the deformation temperature T_d_, at which a certain stress was applied to deform it. Then, the temperature was lowered to a fixed T_f_ = 200 °C (T_f_ < T_g_) at 8 °C/min while the stress was kept unchanged. After the stress was removed at T_f_, the spline was heated to T_d_ at 8 °C/min. The strain variation of the spline was recorded throughout the cooling and heating processes.

Shape fixity (R_f_) and shape recovery (R_r_) of the polyimide films were calculated according to the recorded spline deformation following Equations (1) and (2) [17]

(1) $$R_{f}\left(N\right)=\frac{\varepsilon_{f}\left(N\right)}{\varepsilon_{m}\left(N\right)}\times 100\%$$

(2) $$R_{r}\left(N\right)=\frac{\varepsilon_{m}\left(N\right)-\varepsilon_{p}\left(N\right)}{\varepsilon_{m}\left(N\right)-\varepsilon_{p}\left(N-1\right)}\times 100\%$$

where ε*_f_* represents the strain of the spline at the fixed temporary shape after removing the stress, ε*_m_* represents the maximum strain under stress, ε*_p_* represents the residual strain in the sample after the shape memory test, and *N* represents the number of cycles.

## 3. Results and Discussion

### 3.1. Chemical Spectrum Studies

**Figure 1:**
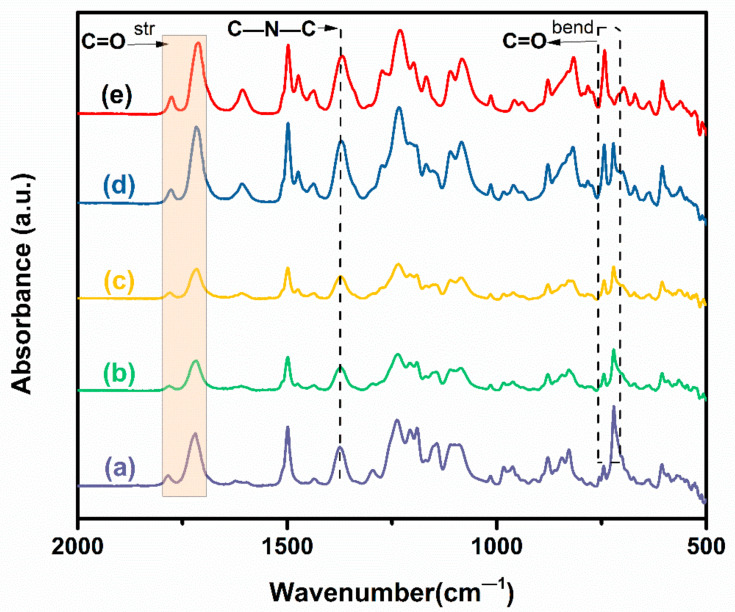
FTIR spectra of PI films of different copolymerization systems: (**a**) 6FDA-ODA, (**b**) 6FDA/ODPA-ODA-73, (**c**) 6FDA/ODPA-ODA-55, (**d**) 6FDA/ODPA-ODA-37, (**e**) ODPA-ODA.

The anticipated polyimide structure was proved with FTIR spectra, as shown in Figure 1. The band at 1775 cm^−1^ ascribed to the carbonyl C=O asymmetric stretching vibration indicates the existence of the imide ring [25], while the band at 1713 cm^−1^ belongs to the carbonyl C=O symmetric stretching vibration, which represents the imide group. The bands around 1370 cm^−1^ correspond to the C-N-C bond, and those between 720 and 740 cm^−1^ originate from the bending vibration of C=O. There was no C=O stretching vibration band for the amide group near 1655 or 1542 cm^−1^, indicating that the imidization was complete.

### 3.2. Thermomechanical Properties of PI Films

**Figure 2:**
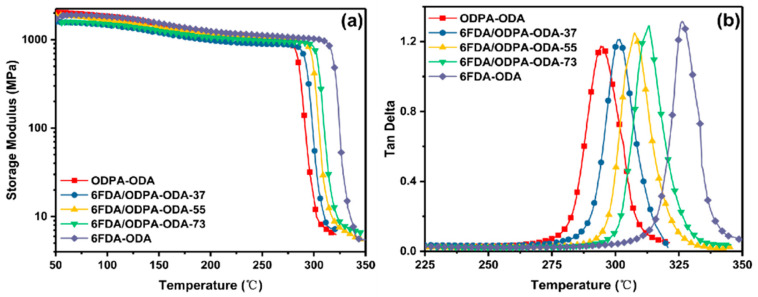
(**a**) Storage modulus and (**b**) tan delta of PI films of different copolymerization systems.

The DMA data of the PI films prepared in this study are summarized in Figure 2 and Table 1. In the glass transition process, the storage modulus of all the five PI film samples dropped sharply from 103 MPa (glassy state) to 10 MPa (rubbery state) (Figure 2a). The rapid decrease in the storage modulus is conducive to better fixing the temporary shape and improving shape fixity. Figure 2b suggests an increase in the temperature corresponding to the peak value of the tan delta along with the increasing 6FDA content, i.e., a gradual increase in the films’ glass transition temperature. The 6FDA-ODA system exhibits the most intense tan delta peak, which is indicative of the rapid fixation of the sample shape at low temperatures [26], namely a high shape fixity.

Table 1 lists the measured glass transition temperature (T_g_) of the PI films of the five systems, the T_g_ values calculated according to the Fox equation, and the storage modulus at 20 °C above the glass transition temperature (E’(T_g_ + 20 °C)). The T_g_ of the five systems ranges from 294 °C to 326 °C. The relationship between the proportion of the two components and the glass transition temperature basically conforms to the Fox equation [27]. Since both ODPA and ODA contain a large number of flexible ether bonds, the chain segments move more easily when the temperature increases [28]. Therefore, a higher ODPA content means a lower glass transition temperature.

The storage modulus of PI films in the rubber state is between 5.4 and 9 MPa, and E’(T_g_ + 20 °C) in the rubber state of ODPA-ODA, which reaches 8.89 MPa, is slightly higher than those of the other four systems. According to the rubber elasticity theory [29], the higher storage modulus is due to the higher crosslink density in the system. The ODPA-ODA system contains a large number of ether bonds, and the molecular chain is more flexible and prone to entanglement, so the system may have a greater physical crosslinking density. This physical crosslinking behavior of the molecular chain helps the ODPA-ODA system store more elastic energy in the process of shape memory, giving the PI film a larger shape recovery force and a higher shape recovery.

### 3.3. Shape Memory Studies

#### 3.3.1. Shape Memory Performance of PI Films of Different Systems

The principle of the shape memory properties of polyimides lies essentially in the transformation between the reversible phase and the fixed phase. At temperatures higher than T_g_, the molecular segments in the fixed phase remain fixed due to the presence of physical crosslinks, while the molecular segments in the reversible phase begin to move. In this case, the film will deform under a stress. Removing the stress at a lower temperature will fix the molecular segments in the reversible phase to reach a temporary shape. Then, when re-heating the film over T_g_, the molecular segments in the reversible phase begin to move. The fixed phase remains fixed, while the molecular segments gradually return to the thermal equilibrium state, which is macroscopically manifested as the shape recovery of the PI film. To eliminate the influence of residual stress in the PI film during preparation, the shape fixity and shape recovery of PI films were calculated during the second heating cycle.

**Figure 3:**
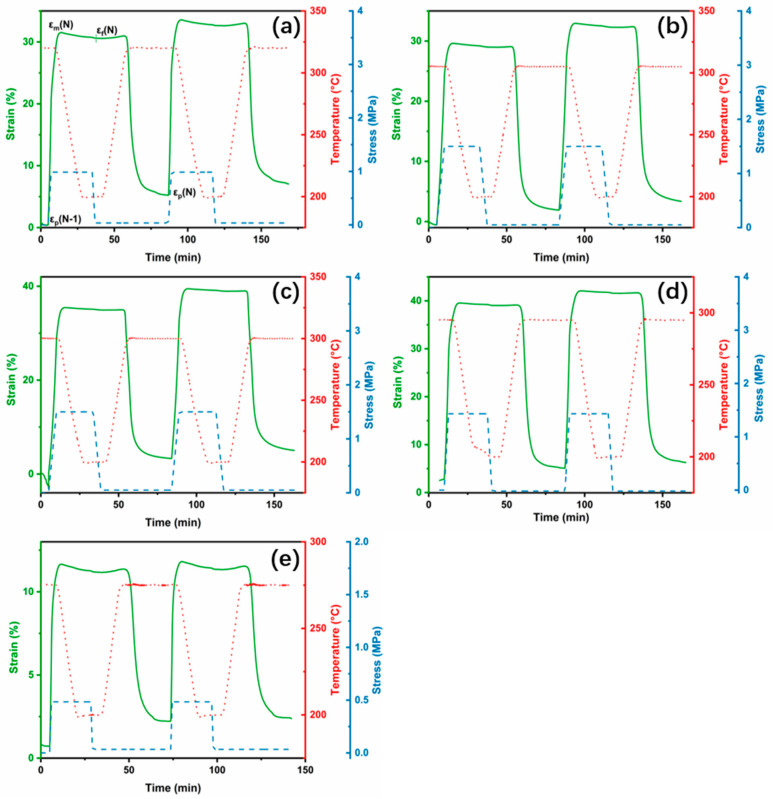
Shape memory properties of (**a**) 6FDA-ODA, (**b**) 6FDA/ODPA-ODA-73, (**c**) 6FDA/ODPA-ODA-55, (**d**) 6FDA/ODPA-ODA-37, (**e**) ODPA-ODA PI films at T_d_ = T_g_ and T_f_ = 200 °C. Solid line: strain; dotted line: temperature; dashed line: stress.

The shape behavior, shape fixity, and shape recovery of PI films of different systems are depicted and gathered in Figure 3 and Table 2, respectively. The 6FDA-ODA system shows a higher R_f_, 97.32%, but its R_r_ is the lowest among the five systems, only 93.60%. In comparison, the ODPA-ODA system leads to the lowest R_f_ and the highest R_r_. The R_f_ of the copolymer systems is significantly higher than in those of the two neat systems, and R_r_ increases with the increasing ODPA content in the system.

**Figure 4:**
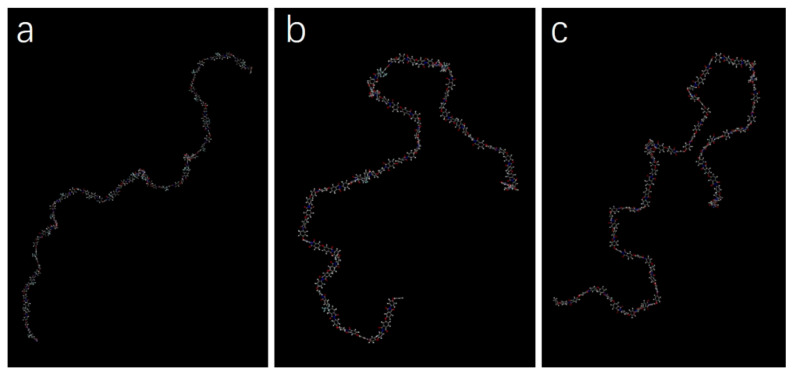
Three-dimensional illustration of molecular structures of shape memory polyimide (**a**) 6FDA-ODA, (**b**) 6FDA/ODPA-ODA-37, (**c**) ODPA-ODA.

The 3D structures of 6FDA-ODA, 6FDA/ODPA-ODA-37, and ODPA-ODA are calculated by Material Studio’s molecular dynamics simulation, as presented in Figure 4, and the architecture diagrams of the others are shown in the supporting information. It can be seen that increasing the ODPA component leads to a larger bending degree of the molecular chain and higher flexibility, which can explain to a certain extent the differences in fixity and recovery amounts within various PI systems. A 6FDA-ODA system has less flexibility, a smaller degree of entanglement between molecular chains, and lower physical crosslinking density, which makes the film slip easily. As the center of mass moves, the material is prone to permanent deformation after drawing, leading to its low recovery rate. The ODPA-ODA system contains a large number of ether bonds in its molecular structure, and the molecular chain has higher flexibility, stronger movement ability, and a greater tendency to entangle [29,30]. When drawing at high temperatures, the chain segment is easy to move and deform, which stores elastic energy when deviating from the equilibrium position. However, part of the ODPA-ODA system will recover under the action of elastic energy when the external force is removed, resulting in low shape fixity. In addition, a low glass transition temperature and an easy entanglement make it possible to complete the entropy elastic recovery at a lower energy state. With the molecular chain returning to the equilibrium position, the film returns to the initial state, showing a high shape recovery.

On the basis of the structural characteristics and shape recovery features of the above two system films, we designed and prepared a 6FDA/ODPA-ODA ternary copolymer system PI film to study its shape memory behavior. With the increase in ODPA content, the shape recovery of the PI film rises from 95.28% to 96.77% (Table 2). This is mainly because the addition of ODPA makes the system more flexible. The molecular chains are more prone to entanglement, which raises the physical crosslinking density and thereby benefits the shape recovery of PI film. It is particularly noteworthy that the curves in Figure 3b–d, corresponding to the cooling setting of films after high-temperature deformation, are relatively straight without small fluctuations in instantaneous spring back, such as those displayed by the 6FDA/ODA and ODPA/ODA systems. This suggests the excellent shape fixing ability of the copolymerized films, consistent with the data in Table 2.

#### 3.3.2. Cyclic Test of Shape Memory Properties of Copolymer PI Films

**Figure 5:**
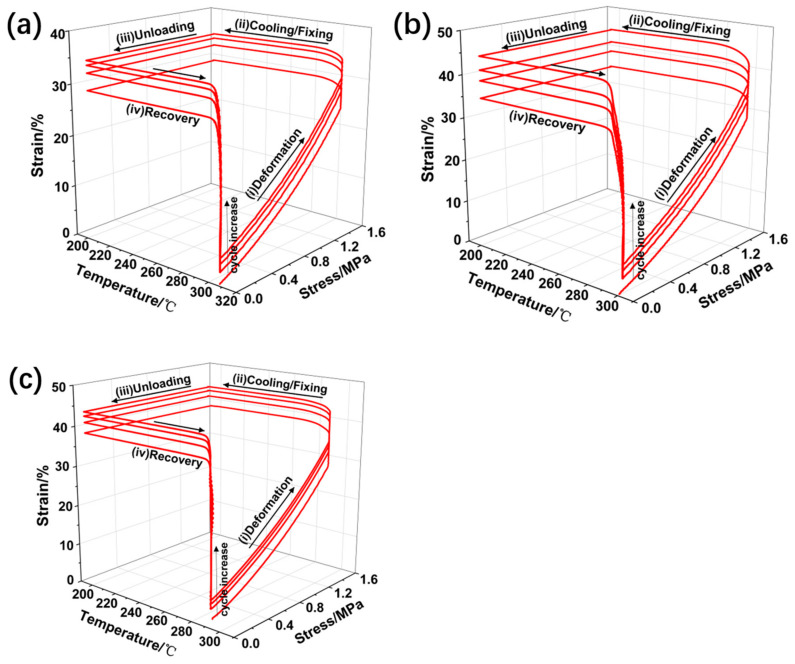
Shape memory properties cycles of (**a**) 6FDA/ODPA-ODA-73, (**b**) 6FDA/ODPA-ODA-55, and (**c**) 6FDA/ODPA-ODA-37 PI films at T_d_ = T_g_, T_f_ = 200 °C.

Shape memory cycle experiments were performed on the 6FDA/ODPA-ODA-73, 6FDA/ODPA-ODA-55, and 6FDA/ODPA-ODA-37 copolymers. As shown in Figure 5, cyclic testing causes increased strains for all the three systems, and the difference in the strain variable after four cycles is less than 10%. The strain increment might derive from the slight shift in molecular chains’ center of mass after each cycle.

**Figure 6:**
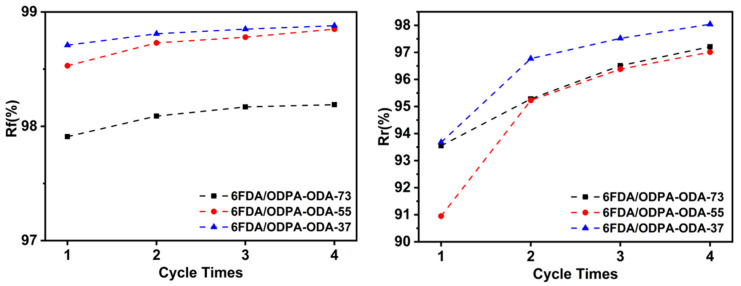
The shape fixity and shape recovery of different copolymerization systems.

Shape fixity and shape recovery of the three copolymer systems are compared in Figure 6. The data in shape memory cycles can be found in the Table S1. As the number of cycles increases, shape fixity remains at a relatively high level while the shape recovery increases. Specifically, 6FDA/ODPA-ODA-37 exhibits the best shape memory performance, with R_f_ and R_r_ reaching 98.88% and 98.04%, respectively, after cycling, which achieves a high level in thermoplastic systems. The improved shape memory performance is due to the fact that the PI molecular chains are stretched during cycling and align along the drafting direction. As they come closer to each other, π–π interactions are more likely to occur, and more physical crosslinking points tend to be formed [24]. Consequently, shape recovery performance improves.

#### 3.3.3. Shape Fixation and Folding—Recovery Properties of Copolymerized PI Films

**Figure 7:**
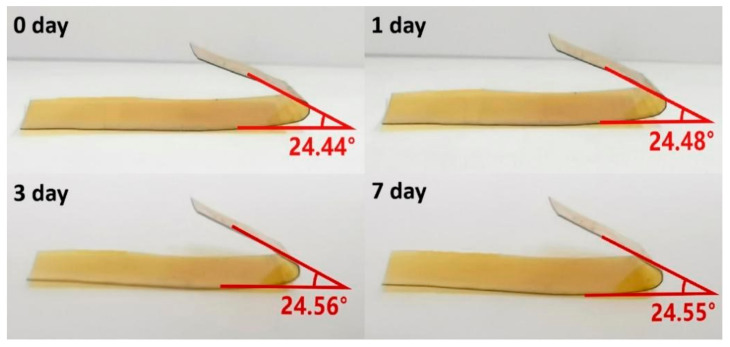
Shape fixing property of 6FDA/ODPA-ODA-37 PI film.

The 6FDA/ODPA-ODA-37 system was further subjected to shape fixation testing. The PI film was first deformed at a high temperature and then fixed at room temperature for shape observation. Figure 7 records its deformation state at 0 days, 1 day, 3 days, and 7 days. Its deformation state hardly changed over time, indicating an excellent shape fixing ability.

**Figure 8:**
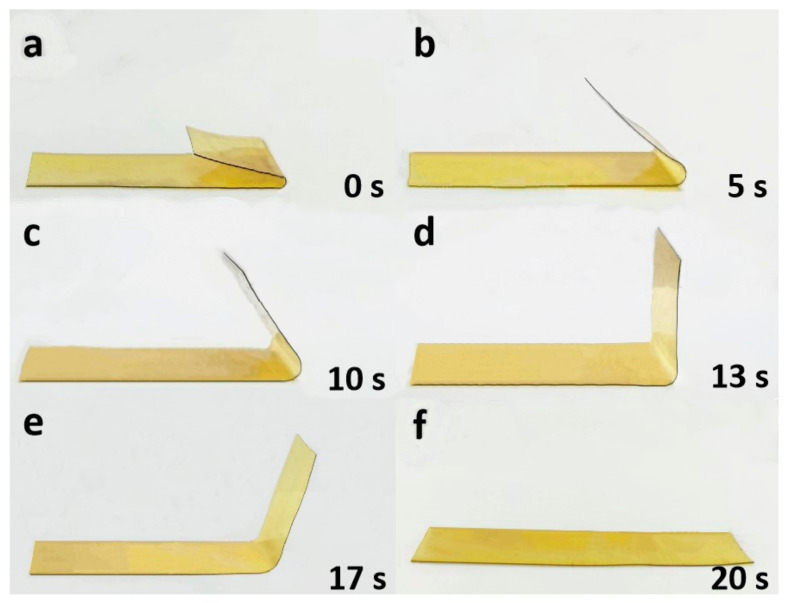
Folding–restoring shape memory properties of 6FDA/ODPA-ODA-37 PI film. (**a**) Temporary shape; (**b**–**e**) Shape recovery when heating at 320 °C; (**f**) Recovered origin shape.

For folding–recovery testing, the hot plate was first heated to a deformation temperature of 320 °C (T_d_ = T_g_ + 20 °C) and held there for 15 min. Next, a piece of 6FDA/ODPA-ODA-37 film was placed on the plate for even heating, then deformed and transferred away from the plate for shape fixation at room temperature. Later on, the film was replaced on the hot plate at 320 °C for shape recovery observation. The photographs at the recovery times of 0 s, 5 s, 10 s, 13 s, 17 s, and 20 s, are captured in Figure 8. After being folded to nearly 180°, the PI film returns to its initial flat shape in about 20 s. Moreover, repeated folding and recovery hardly deteriorate its shape memory performance, evidencing excellent recycling characteristics of the shape memory PI film prepared.

### 3.4. Thermal Stability and Mechanical Properties of PI Films

**Figure 9:**
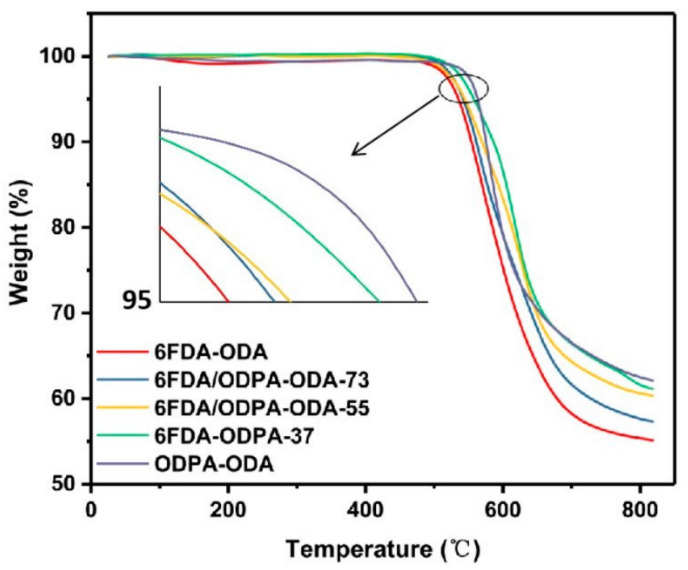
TG curves of PI films of different copolymerization systems.

Favorable thermal and mechanical properties are key factors that support the broad application of shape memory polyimide. The temperature at 5% weight loss (T_5_) was used to characterize copolymer’s thermal stability. The thermogravimetric (TG) curves are plotted in Figure 9, with the T5 values collected in Table 3. All five copolymerization systems achieved T_5_ over 535 °C. Among them, T_5_ of the 6FDA-ODA system is the lowest (535.31 °C) while that of the ODPA-ODA system is the highest (563.36 °C); a synergistic growth of T_5_ with the increasing proportion of ODPA is clearly noted. In addition, the residual weight of all the PI films at 800 °C is more than 55%, indicating that these shape memory PI films have excellent thermal stability.

**Figure 10:**
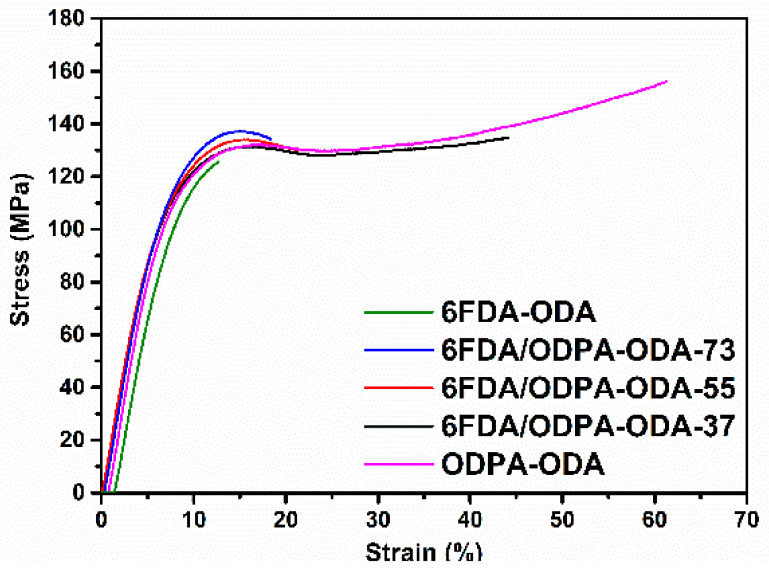
Stress—strain curve of PI films with different systems.

Listed in Table 3 are the mechanical properties of the PI films. Each value is averaged after five tests. The values in brackets are standard deviations, indicating that the mechanical properties of the film have good stability. The stress–strain curve of PI films with different systems is shown in Figure 10. With more ODPA incorporated into the system, the elongation at break gradually increased from 15.11% to 66.2%. This can be explained by the enhanced flexibility of molecular chains due to more ether bonds introduced by the addition of ODPA, which conduces to chain entanglement and thereby an improvement in the film’s toughness. All the shape memory films exhibit excellent mechanical properties, of which the modulus and tensile strength are higher than 2 GPa and 125 MPa.

## 4. Conclusions

In summary, a series of high-temperature shape memory PI films were prepared via facile random copolymerization of ODA with 6FDA and ODPA. Their thermomechanical performance, shape memory behaviors, and mechanical properties were characterized and analyzed to discuss the influence of dianhydride proportion on the structure and properties of the film. Results indicate that the shape fixity rate and shape recovery reached 98.81% and 96.77%, respectively, by carefully tuning the ratio of 6FDA and ODPA in the copolymer system. After many fixing–recovery cycles, the maxima R_f_ and R_r_ of 98.88% and 98.04%, respectively, were obtained, which proved excellent shape memory performance for the PI films. All the films exhibit excellent thermal and mechanical properties when the thermal decomposition temperature is above 535 °C, and modulus and tensile strengths are higher than 2 GPa and 125 MPa. The polyimide prepared in this work is expected to be applied in the field of space expandable structures under harsh environments.

## Figures and Tables

**Table 1:** E’(T_g_ + 20 °C) and T_g_ of PI films of different copolymerization systems.

Sample	T_g_ (°C)		E’ (MPa) at T_g_ + 20 °C
	DMA	Fox Equation	
6FDA-ODA	326.72	326.72	5.44
6FDA/ODPA-ODA-73	312.85	316.37	7.43
6FDA/ODPA-ODA-55	307.25	309.83	7.19
6FDA/ODPA-ODA-37	301.66	303.55	7.59
ODPA-ODA	294.60	294.6	8.89

**Table 2:** R_f_ and R_r_ of PI films of different copolymerization systems.

Sample	R_f_(%)	R_r_(%)
6FDA-ODA	97.32	93.60
6FDA/ODPA-ODA-73	98.09	95.28
6FDA/ODPA-ODA-55	98.73	95.23
6FDA/ODPA-ODA-37	98.81	96.77
ODPA-ODA	96.16	98.33

**Table 3:** Thermal and mechanical properties of PI films of different copolymerization systems.

Sample	T_5_ (°C)	Tensile Strength (MPa)	Elongation at Break (%)	Modulus (GPa)
6FDA-ODA	535.31	125.17(7.29)	15.11(0.19)	1.98(0.043)
6FDA/ODPA-ODA-73	542.13	133.96(3.79)	18.34(4.18)	2.03(0.089)
6FDA/ODPA-ODA-55	544.51	131.86(4.03)	20.65(1.06)	2.06(0.021)
6FDA/ODPA-ODA-37	557.83	133.13(2.47)	39.44(6.66)	2.02(0.037)
ODPA-ODA	563.36	157.62(5.07)	66.2(2.43)	2.07(0.059)

## Data Availability

The data presented in this study are available within the article.

## Supplementary Materials

The following are available online at https://www.mdpi.com/article/10.3390/polym13193222/s1, Figure S1: T_g_ of PI films of different copolymerization systems, Figure S2: 3D illustration of molecular structures of shape memory polyimide, Table S1: The R_f_ and R_r_ data in shape memory cycles (in Figure 6).
