# Supplementary material for: High-Temperature-Induced Shape Memory Copolyimide
Source: Polymers (Basel). 2021 Sep 23;13(19):3222. doi: 10.3390/polym13193222 (PMC8512334; doi:10.3390/polym13193222)
Supplement: Supplementary file 1 [file polymers-13-03222-s001.zip › polymers-1371478-supplementary.pdf]

Supplementary Information

High-temperature-induced Shape Memory Copolyimide

Supplementary information

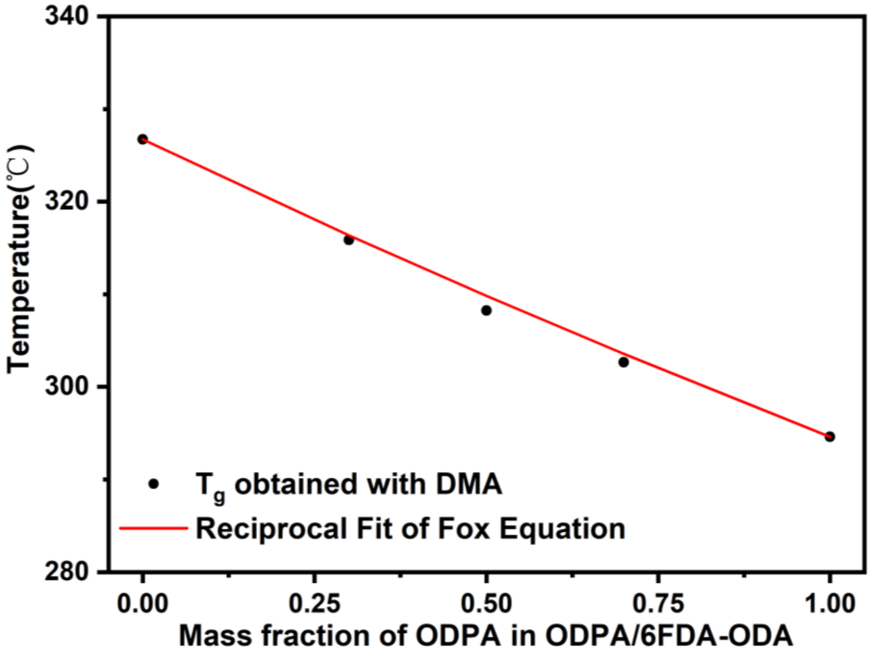

Figure S1. T_g of PI films of different copolymerization systems.The red line is calculated according to the Fox equation.The points are tested by DMA.

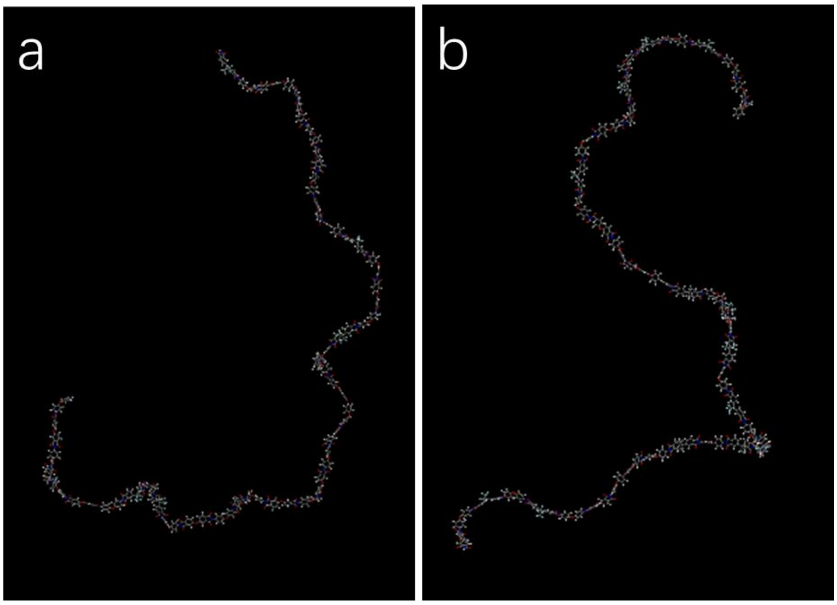

Figure S2 3D illustration of molecular structures of shape memory polyimide (a) 6FDA/ODPA-ODA-55 (b) 6FDA/ODPA-ODA-73.

Table S1 The R_f and R_r data in shape memory cycles (in Figure. 6)

Sample	Cycle	R_f (%)	R_r (%)
6FDA/ODPA-ODA-73	1st	97.91	93.55
	2nd	98.09	95.28
	3rd	98.17	96.51
	4th	98.19	97.21
6FDA/ODPA-ODA-55	1st	98.53	90.95
	2nd	98.73	95.23
	3rd	98.78	96.38
	4th	98.85	97.01
6FDA/ODPA-ODA-37	1st	98.71	93.68
	2nd	98.81	96.77
	3rd	98.85	97.52
	4th	98.88	98.04
